# Prediction and Optimization of Surface Roughness in a Turning Process Using the ANFIS-QPSO Method

**DOI:** 10.3390/ma13132986

**Published:** 2020-07-04

**Authors:** Mahdi S. Alajmi, Abdullah M. Almeshal

**Affiliations:** 1 Department of Manufacturing Engineering Technology, College of Technological Studies, The Public Authority for Applied Education and Training, Safat 13092, Kuwait; 2 Department of Electronic Engineering Technology, College of Technological Studies, The Public Authority for Applied Education and Training, Safat 13092, Kuwait; AM.Almeshal@paaet.edu.kw

**Keywords:** adaptive neuro-fuzzy inference system, turning process, surface roughness, machine learning, quantum particle swarm optimization, ANFIS-QPSO, ANN

## Abstract

This study presents a prediction method of surface roughness values for dry and cryogenic turning of AISI 304 stainless steel using the ANFIS-QPSO machine learning approach. ANFIS-QPSO combines the strengths of artificial neural networks, fuzzy systems and evolutionary optimization in terms of accuracy, robustness and fast convergence towards global optima. Simulations revealed that ANFIS-QPSO results in accurate prediction of surface roughness with RMSE = 4.86%, MAPE = 4.95% and R^2^ = 0.984 for the dry turning process. Similarly, for the cryogenic turning process, ANFIS-QPSO resulted in surface roughness predictions with RMSE = 5.08%, MAPE = 5.15% and R^2^ = 0.988 that are of high agreement with the measured values. Performance comparisons between ANFIS-QPSO, ANFIS, ANFIS-GA and ANFIS-PSO suggest that ANFIS-QPSO is an effective method that can ensure a high prediction accuracy of surface roughness values for dry and cryogenic turning processes.

## 1. Introduction

In the turning process, surface roughness performs a vital role in the creation of products, and also exerts great influence on machining cost because it is considered an index of product quality [1]. However, surface roughness defines such mechanical properties as corrosion, wear, lubrication, electrical conductivity and fatigue behavior [2]. Moreover, the surface roughness of any machining process has become prominent because of the heightened quality demands. The production of a desired surface finish on a piece of work is mainly affected by machining parameters, such as cutting speed, feed rate, depth of cut, tool geometry, workpiece material and other factors such as tool wear, vibrations, machine dynamics and temperature. Meanwhile, heat is generated during the turning process and the uses of cutting fluid provide lubrication and cooling, which affects and progresses the final quality of the workpiece. Cutting fluids improve the efficiency of machining in terms of improved surface finish, improved dimensional accuracy, reduced tool wear and reduced cutting temperature. Sen et al. [3] presented the advance in capabilities of the ecofriendly minimum quantity lubrication (MQL) technique. The authors discussed the advantages of MQL and illustrated a review of literature of MQL assisted machining operations. Rapeti et al. [4] use the application of vegetable oil based nano cutting fluids (coconut oil, sesame oil and canola oil) during the turning of AISI 1040 steel. Economic analysis for the application of nano cutting fluids is done to assess the viability of these fluids in the industry. Kanth et al. [5] investigated the use of a mixture of nano crystalline graphite and sunflower oil as an alternative for cutting fluids for an improved surface roughness finish in a turning process. The study revealed that sunflower oil results in a better surface roughness finish when compared to other vegetable oils.

In the past, machining parameters used to be selected by the trial and error that was time consuming and costly, based on process planners’ experience and machining handbooks [6]. A human process planner chooses proper machining process parameters that depend on his own experience or his machining tables. In most cases, the selected parameters are conventional and far from optimal. However, in machining it is significant to choose the proper parameters. If the machining parameters are not appropriate, excessive cutting tool wear is noticed and the choice may result in surface damage.

Surface roughness refers to the shape of the surface to be machined and combined with surface quality. The appearance of the surface roughness mechanism is very complex and mostly depends on highly analytical equations. The surface finish can be characterized by two main parameters, average roughness ($R_{a}$) and maximum peak to valley height ($R_{t}$). Theoretical models have been used to calculate these parameters [7]. A basic theoretical model for surface roughness is given by Equation (1)

(1) $$R_{a}=\frac{1000f^{2}}{32r_{e}}$$

(2) $$R_{t}=\frac{1000f^{2}}{8r_{e}}$$

where f is the feed rate and $r_{e}$ is the tool nose radius. Based on this model, one need only reduce the feed rate or increase the tool nose radius to produce the desired surface roughness. This model to some extent presumes a large nose radius and a slow feed. For a zero nose radius and a somewhat larger feed, the following model is suggested by Boothroyd and Knight [8]

(3) $$R_{a}=\frac{f}{4\left(\cot\;\left(\alpha \right)+\cot\left(\beta \right)\right)}$$

where α and β are the major and end cutting edge angles respectively, and cot is the cotangent function. Fang and Safi-Jahanshahi [9] present linear and exponential empirical models for surface roughness as functions of cutting speed (V), feed (f) and depth of cut (d):

(4) $$R_{a}=C_{0}V^{a}f^{b}d^{c}$$

where C is constant and a, b and c are the exponents.

In the present paper, empirical models are established with conventional methods such as a factorial design, statistical regression and response surface methodology. Artificial intelligence-based models are introduced using nonconventional approaches such as the artificial neural network (ANN), fuzzy logic (FL), support vector regression (SVR) and a genetic algorithm (GA) [10]. Using conventional methods may not be enough to define the nonlinear complex relationship between machining parameters and machining performance. Lately a good deal of attention has been devoted to establishing predictive and optimization models in order to consider the effect of machining parameters on machining functioning, using artificial intelligence methods as an alternative to conventional methods. Trung-Thanh Nguyen [11] applies a microgenetic algorithm (AMGA) for dry milling in order to resolve the trade-off analysis between the material removal rate, specific cutting energy and surface roughness. Camposeco-Negrete [12] uses a robust design technique to control the results and contributions of four machining parameters on the above-mentioned response variables in wire-cut EDM. Soepangkat et al. [13] propose a grey fuzzy analysis and BPNN-based GA to control and predict the optimal parameters in the drilling KFRP. Venkata and Murthy [14] combine predictive models such as response surface methodology, artificial neural networks and support vector machine to predict the surface roughness and root mean square of work piece vibration in the boring process. Prasath et al. [15] developed a mathematical model for prediction response employing Taguchi and response surface methodology (RSM). The model is confirmed and predicted the surface roughness and MRR with less than 6% of error. Matras et al. [16] introduced new optimized method that involves the prediction of the curvilinear surface roughness in turning titanium alloy. The created model also results in a short machining time and low manufacturing cost. The machining time was significantly reduced in comparison to the non-optimized cutting process. Mia and Dhar [17] presented a prediction model development of surface roughness in hard turning when the experimental runs were conducted under both dry and high-pressure coolant (HPC) conditions. the prediction model was prepared by employing support vector regression (SVR) and response surface methodology (RSM) and the optimization model was constructed by embracing the composite desirability function (CDF) and genetic algorithm (GA). The predictive model by SVR and optimization model by GA provided the highest accuracy. Yadav [18] applied a hybrid approach of the Taguchi methodology-response surface methodology (TM-RSM), which has been implemented for modeling and optimization for the duplex turning process. The optimum condition obtained from TM has been used as a central value in RSM for the modeling and optimization. The result shows the significant improvement in surface finish with the hybrid approach as compared to the Taguchi analysis. Chabbi et al. [19] investigated the influence of cutting parameters on the finish of surface roughness during the cutting of the polyoxymethylene (POM C) by utilizing the response surface methodology (RMS) method. The results revealed that the surface roughness was strongly influenced by the feed rate with a large contribution, followed by the cutting depth, whereas, the cutting speed has no influence. A recent study on the use of dry, mono-jet and dual-jet of cryogenic conditions in the turning process was presented in [20]. The Taguchi full factorial orthogonal array design was used to study the machining responses of Ti-6Al-4V alloy and grey relational analysis (GRA) method has been utilized to optimize the parameters. The results illustrated that ideal responses can be achieved using the dual-jet LN2 cryogenic condition.

Other soft computing machine learning approaches, such as ANFIS, have been proposed to predict workpiece surface roughness in the turning operation. Jain and Raj [21] introduce monitoring systems that use ANFIS to predict the surface roughness. This model shows the ability to estimate tool life for an unmanned manufacturing system related to surface roughness. Elbaz et al. [22] propose a model based on the fuzzy C-mean (FCM) clustering method that combines enhanced particle swarm optimization (PSO) with ANFIS. The computational model was used to predict the performance of an earth pressure balance (EPB) shield during tunneling. The prediction results indicate an accurate prediction of the EPB and good agreement between the actual measurements and the predicted values. Zhang et al. [23] develop two computational models based on the random forest (RF) algorithm. A hybrid algorithm PSO-RF is proposed to optimize operational parameters in real time during the tunneling process so that tunneling-induced settlement can be controlled within the tolerated values. The results demonstrate that the predicted results are accurate when compared with actual settlements. Chen et al. [24] apply three artificial neural network (ANN) methods: back-propagation (BP), a neural network (the radial basis function (RBF) neural network) and the general regression neural network (GRNN) to predict the maximum surface settlement caused by EPB shield tunneling. The results of analysis show that close correlations were established between the predicted and the measured settlements in the GRNN model with MAE = 1.10, and RMSE = 1.35, respectively. Shivakoti et al. [25] present predictions about the machining of stainless steel 202, based on the adaptive network-based fuzzy inference system and parametric analysis of CNC lathe-process parameters. The experimental outcomes and ANFIS predicted results are compared, confirming the precise prediction of ANFIS outcomes in the course of turning stainless steel 202. Maheshwera et al. [26] analyze the influence of machining parameters on surface roughness by establishing regression analysis (RA) and artificial neural network (ANN) models during the turning of hard work material, AISI 52,100 steel. The prediction performance of the ANN model is shown to be better than that of the RA model and is expected to be a practical way of reducing the required time and expense of experimental runs. Palanisamy and Senthil [27] introduce an adaptive neuro fuzzy inference system (ANFIS) to define the relationship between the count input machining conditions and output measures such as the cutting force and surface roughness of the machined surface. The achieved results reveal the development of output quality combined with lower production cost, which is evident of the efficiency of the established ANFIS model. Arapoglu et al. [28] suggested new variable selection method based on artificial neural networks (ANN) for the prediction of the surface roughness. A statistical hypothesis test is used as an elimination criterion. The selection of variables does not change the prediction accuracy of the model at the 1% significance level.

The reported literature suggests that machine learning approaches, such as ANN and ANFIS, have shown efficacy in predicting the machining parameters of various applications. When compared to ANFIS, a hybrid ANN approaches such as ANN-RBF and ANN-BPFN have a more complex structure and require high computation power. In addition, the hybridization of ANFIS with evolutionary algorithms such as GA and BFA would require more computational time due to the nested populations in the GA and BFA algorithms. In addition, ANFIS-PSO have shown it to be effective in predicting surface roughness but ANFIS-PSO may not converge to global optima and could get trapped in local optima [29]. QPSO, however, has been found highly effective, outperforming PSO in several applications due to its simple implementation and fast global optimum convergence [29].

In this research, we propose the use of ANFIS-QPSO for predicting the surface roughness in the turning process. To the best of our knowledge, there is a gap in literature in utilizing ANFIS-QPSO to predict surface roughness in the turning process. In addition, no previous study has investigated the use of the ANFIS-QPSO approach for predicting the surface roughness in dry and cryogenic turning processes. The present study set out to examine the accuracy of ANFIS-QPSO in predicting the experimental dataset of a dry and cryogenic turning process involving AISI 304 stainless steel. In the next section, the methodology of the proposed ANFIS-QPSO approach is presented with nomenclature presented in Table 1. The simulation results present the predicted results and highlight the prediction accuracy of ANFIS-QPSO when compared with the classical ANFIS approach.

## 2. Methodology

### 2.1. Adaptive Neuro-Fuzzy Inference System (ANFIS)

ANFIS is a hybrid intelligent computing approach that combines artificial neural networks with a fuzzy system for various applications, including system identification, parameter prediction and energy load forecasting [30,31,32,33,34]. ANN is a soft computing method that mimics the human brain and is defined by interconnected layers consisting of processing neuros or nodes. ANN consists of input layers, hidden interconnected layers and an output layer. ANN can learn the pattern of data via applying at each node simple calculations, consisting of the multiplication of weights and the addition of bias.

**Figure 1:**
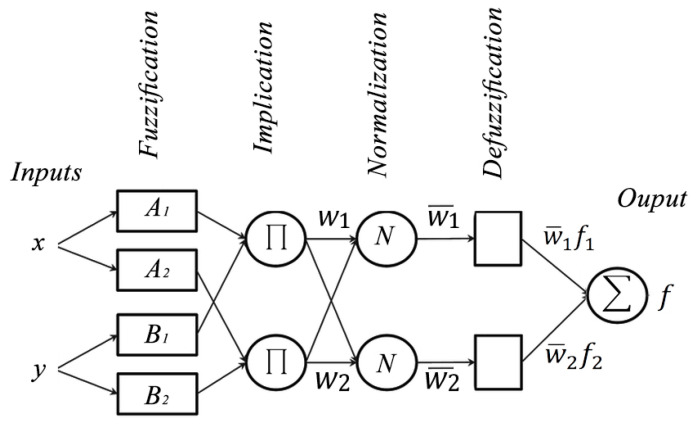
ANFIS structure.

A fuzzy system is a control approach that is based on fuzzy logic. Fuzzy logic maps nonlinear input data into scalar outputs via a series of if-then rules based on human experiences. Fuzzy systems consist of a series of processes involving fuzzification, a fuzzy inference engine and defuzzification. This process transforms crisp values into a corresponding linguistic fuzzy variable to be fed into a fuzzy inference engine. Fuzzy systems have two system models, the Takagi-Sugeno fuzzy system model and a Mamdani fuzzy system model. The fuzzy inference engine applies the fuzzy rules to the fuzzy variables via implication operations. The outputs are then fed into the defuzzification process, where the fuzzy outputs are converted back into crisp values. Fuzzy systems are model free and can be proposed for several applications so long as proper fuzzy rules are designed that are based upon human experiences. In addition, fuzzy systems are robust to disturbances and have shown robust stability despite model uncertainties [35,36,37]. The Takagi-Sugeno fuzzy system model was used in this research. The ANFIS structure is demonstrated in Figure 1. Takagi-Sugeno fuzzy rules are of the following form:

Rule i:

(5) $$If\;x\;is\;A_{i}\;and\;y\;is\;B_{i}\;then\;f_{i}=p_{i}x+q_{i}y+r_{i}$$

The Takagi-Sugeno ANFIS structure consists of five feed-forward layers. These layers are as follows:

#### 2.1.1. Layer 1: Fuzzification Layer

This layer converts all crisp inputs into fuzzy inputs; assuming an ANFIS system with two inputs, x and y with Ai and Bi, representing respectively fuzzy sets for the inputs, the two node outputs can be written as

(6) $$O_{1,i}=\mu_{Ai}\left(x\right),\;\;\;\;i=1,2$$

(7) $$O_{1,i}=\mu_{Bi}\left(y\right),\;\;\;\;i=1,2$$

where $\mu_{Ai}\left(x\right)$ and $\mu_{Bi}\left(y\right)$ are the membership” functions of a Gaussian type that can be represented as:

(8) $$\mu_{Ai}\left(x\right)=e^{-\left(\frac{{\left(c_{i}-x\right)}^{2}}{2\sigma_{i}^{2}}\right)}$$

where $c_{i}$ and $\sigma_{i}$ present the premise parameters set and consist respectively of the mean as well as the standard deviation of a Gaussian function.

#### 2.1.2. Layer 2: Implication Layer

The implication layer calculates the weight functions of the neural network where each node represents a rule firing strength that is described by:

(9) $$O_{2,1}=w_{i}=\mu_{Ai}\left(x\right)\;\;\;\;\Delta \;\;\;\;\mu_{Bi}\left(y\right),\;i=1,2$$

#### 2.1.3. Layer 3: Normalization Layer

The weight functions are normalized to present the normalized rule firing strengths, calculated as:

(10) $$O_{3,1}={\bar{w}}_{i}=\frac{w_{i}}{w_{1}+w_{2}},\;i=1,2$$

#### 2.1.4. Layer 4: Defuzzification Layer

With the adaptive nodes, the fuzzy values are converted into crisp values by calculating:

(11) $$O_{4,1}={\bar{w}}_{i}f_{i}={\bar{w}}_{i}\left(p_{i}x+q_{i}y+r_{i}\right)$$

where $\left(p_{i}x+q_{i}y+r_{i}\right)$ are the consequent parameters set.

#### 2.1.5. Layer 5: Output Layer

Each node output can be calculated as:

(12) $$O_{5,1}=\underset{i}{\sum }{\bar{w}}_{i}f_{i}=\frac{\sum_{i}w_{i}f_{i}}{\sum_{i}w_{i}}$$

### 2.2. The Quantum Particle Swarm Optimization Algorithm (QPSO)

QPSO is an evolution-inspired optimization method. It has been widely applied in many applications due to its simplicity of implementation and its computational efficiency. When evaluated with other evolution-inspired optimization algorithms, such as the genetics algorithm (GA) and bacterial foraging algorithm (BFA), QPSO usually results in faster convergence to optimal values due to the simple algorithm pseudocode. This is due to the fact that GA and BFA are generation-based evolutionary optimization algorithms with nested mathematical operations that require more computational power and more time to converge towards optimal solutions.

Unlike classical PSO, QPSO is highly effective in solving optimization benchmark functions with faster convergence and precise search ability within the space of solutions. In addition, the QPSO algorithm is robust in solving unimodal and multimodal benchmark functions and is prone to premature convergence, local minima that may occur when using PSO [37].

Numerous variations of PSO algorithm have been developed and reported in the literature, such as quantum behaved PSO (QPSO) and deep learning-driven PSO [38]. Variations in different performance include avoiding local maxima and convergence times. In this research, we integrate QPSO with ANFIS to predict the surface roughness of a turning process.

Classical PSO starts by randomly distributing the particles within the space of possible solutions with initialized positions and velocities. Each particle calculates the objective function or fitness and defines it as an individual best solution. A global best solution can then be selected as the best fitness value among all the particles. The next step is to update the positions and velocities of the particles to extend the search towards the optimal value until the stopping criteria are met.

In classical PSO, the positions and velocities of the particles were calculated at each iteration and updated to diversify the search space and converge towards the optimal solution. Thus, the trajectory of the movement of particles within the search space is deterministic. However, in quantum mechanics, according to Heisenberg’s uncertainty principle, the velocity and the position of the particle cannot be determined simultaneously and the state of the particle is described by Schrödinger’s wavefunction ψ(x,t). Solving Shrödinger’s equation to obtain the probability density function of the particles’ location in the space, and using Monte Carlo simulation, the position of movement of the particle can be presented as follows:

(13) $$\{\begin{array}{cc} x_{i,j}\left(t+1\right)=p_{i}\left(t\right)+\beta \left|Mbest_{j}\left(t\right)-x_{i,j}\left(t\right)\right|\ln\left(\frac{1}{u}\right) & \;if\;k\geq 0.5\\ x_{i,j}\left(t+1\right)=p_{i}\left(t\right)-\beta \left|Mbest_{j}\left(t\right)-x_{i,j}\left(t\right)\right|\ln\left(\frac{1}{u}\right) & \;if\;k<0.5 \end{array}$$

where
u and kuniform probability distribution parameters in the range [0,1]$x_{i,j}\left(t+1\right)$the position of the i-th particle in the j-th dimension of the spaceβcontraction-expansion coefficient*p*_i_local attractor pointMbestmainstream thought or mean best value

The mean best value is the mean of all individual best solutions of a population and can be evaluated as follows:

(14) $$Mbest_{j}\left(t\right)=\frac{1}{N}\overset{N}{\underset{j=1}{\sum }}p_{g,j}\left(t\right)$$

where *g* represents the index of the best particle in the population. Additionally, the local attractor $p_{i}$ guarantees the convergence of the algorithm and is defined as:

(15) $$p_{i}\left(t\right)=\frac{c_{1}p_{k,i}+c_{2}p_{g,i}}{c_{1}+c_{2}}$$

with $p_{k,i}$ and $p_{g,i}$ representing the pbest and gbest respectively. The pseudocode of the QPSO algorithm is introduced in Algorithm 1.
**Algorithm 1.** QPSO pseudocode.1: Step 1: Setting population size and random initialization of particle positions and velocities.2: **Begin**
3:  **While** optimal solution not reached yet, **do**:4:    **For** each particle *i*5:      Step 2: Update the particles positions using Equation (13)6:      Step 3: Evaluation of particles fitness according to required objective function7:      Step 4: Calculate fitness of each particle (pbest and gbest) using Equation (14)8:      **end for**9:    Step 5: Update *pbest, gbest*, and *p* using Equation (15)10:   **End While**11: **End**

**Figure 2:**
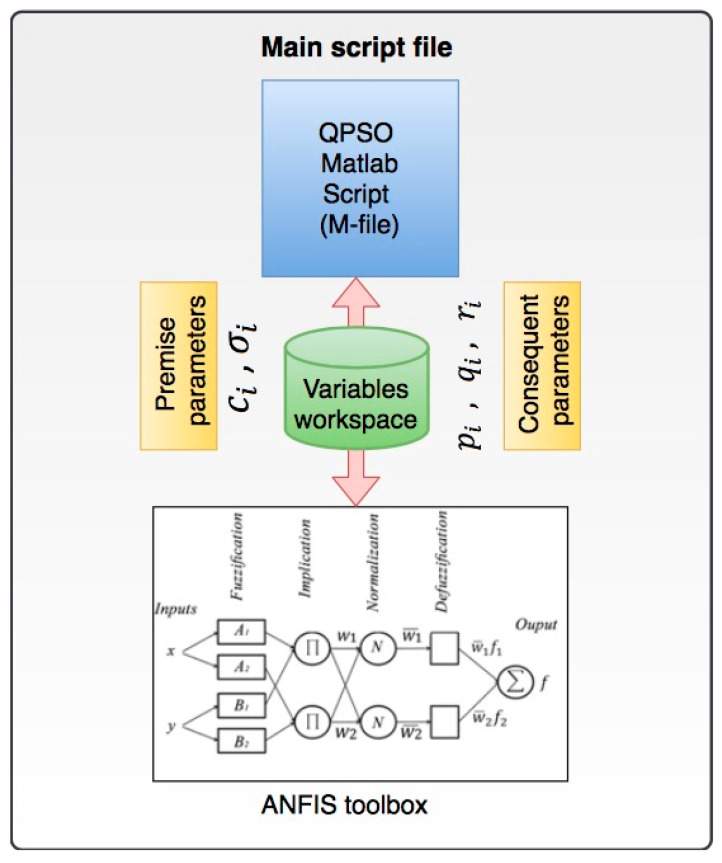
ANFIS-QPSO integration structure.

The QPSO algorithm is utilized to optimize the premise and consequent parameters of the ANFIS system presented in Equations (6), (7) and (11) respectively. This is due to the fact that the classical ANFIS is dependent on backpropagation and gradient descent algorithms for training and learning the membership function parameters. However, gradient descent and backpropagation algorithms are prone to be trapped within local optima and may be unable to converge towards a global optimum solution [39]. As a result, the performance of ANFIS in predicting the outputs would be affected. A proposed solution to overcome this problem and improve the performance of the ANFIS is to integrate it with QPSO, which is independent of the ANFIS structure, and feedback the optimum premise and consequent parameters of the ANFIS layers. The proposed hybrid ANFIS-QPSO architecture is presented in Figure 2. Matlab software package (MathWorks, Natick, MA, USA) was used for the simulation of the ANFIS-QPSO system. The ANFIS system was created with the required structure, membership function types and the number of required inputs and outputs using the ANFIS toolbox in Matlab. Training and testing data proportions were then defined and fed into the ANFIS system. A main script file was then executed to integrate the ANFIS structure with the QPSO algorithm to learn the optimum values of the premise and consequent parameters at each iteration. The process was repeated until the stopping criteria were met, in this case it was defined as the minimum root mean square error (RMSE) value.

In this research, the ANFIS-QPSO algorithm is presented for predicting the surface roughness of cryogenic and dry turning processes. Experimental datasets were split for the training, testing and validation processes. A training split of 70%, testing split of 15% and a validation split of 15% of the experimental dataset were used. In addition, to assess the performance of the model prediction accuracy root mean square (RMSE), the mean absolute percentage error (MAPE) and coefficient of determination (*R*^2^) were adopted as follows:

(16) $$RMSE=\frac{\sqrt{\sum_{i=1}^{n}{\left({\hat{y}}_{i}-y_{i}\right)}^{2}}}{n}$$

(17) $$MAPE=\frac{1}{n}\overset{n}{\underset{i=1}{\sum }}{\left|\frac{{\hat{y}}_{i}-y_{i}}{y_{i}}\right|}^{}\times 100\%$$

(18) $$R^{2}=1-\frac{\sum_{i=1}^{n}{\left(y_{i}-{\hat{y}}_{i}\right)}^{2}}{\sum_{i=1}^{n}{\left(y_{i}-\overset{˘}{y}\right)}^{2}}$$

where y , $\hat{y}$ and $\overset{˘}{y}$ represent the measured, predicted and averaged outputs respectively. In the next section, simulations of ANFIS-QPSO for dry and cryogenic turning processes are presented and compared with the classical ANFIS system to highlight the improvement of the prediction values by the proposed ANFIS-QPSO approach.

## 3. Results

In this section, ANFIS-QPSO will be trained for predicting the surface roughness of the cryogenic and dry turning method with reference to experimental data reported in [40]. The experimental data of the dry and cryogenic turning process are presented in Table 2 and Table 3 respectively, showing 36 trials of each process to evaluate every combination of input parameters of depth of cut, speed and feed rate.

### 3.1. ANFIS-QPSO for Predicting of Surface Roughness of a Dry Turning Process

**Figure 3:**
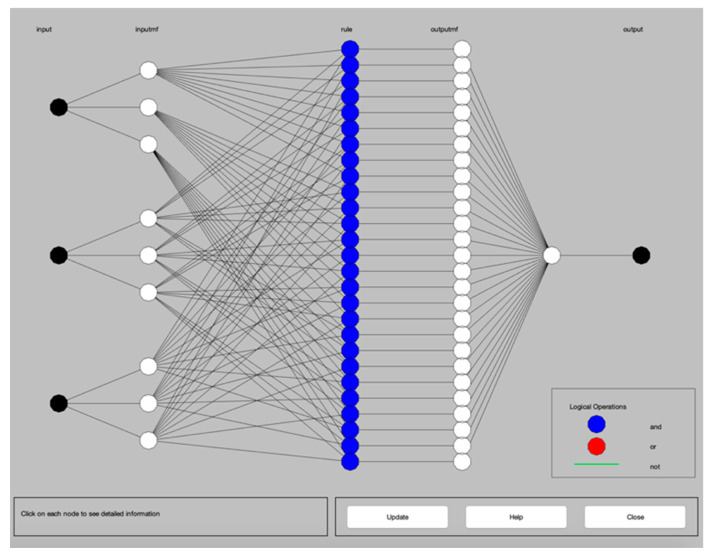
ANFIS structure.

With the dry turning process data of Table 3, the ANFIS-QPSO system was simulated using the parameters presented in Table 4 and the ANFIS structure as illustrated in Figure 3.

**Figure 4:**
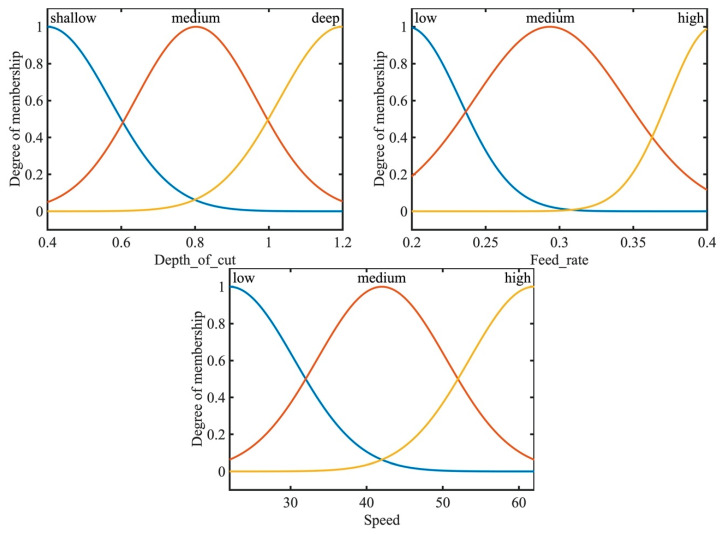
Takagi-Sugeno fuzzy inference engine Gaussian membership functions for the dry turning process.

**Figure 5:**
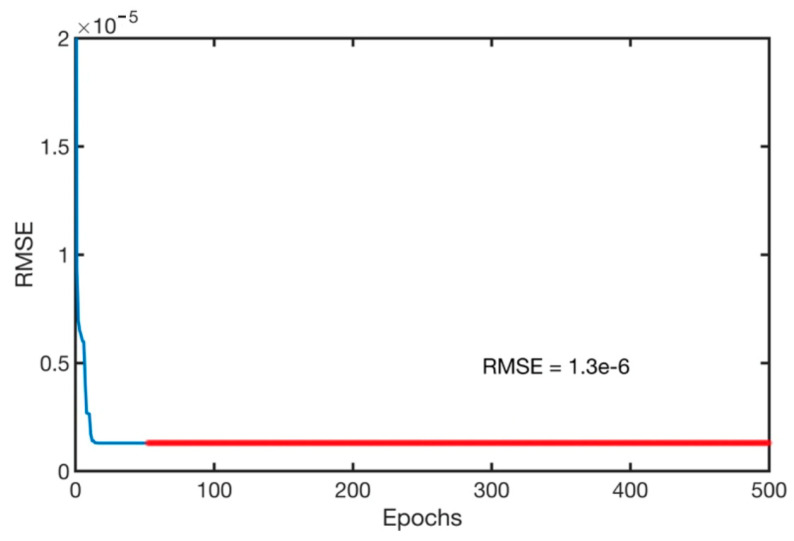
Training epochs vs. root mean square error (RMSE) for the dry turning process.

**Figure 6:**
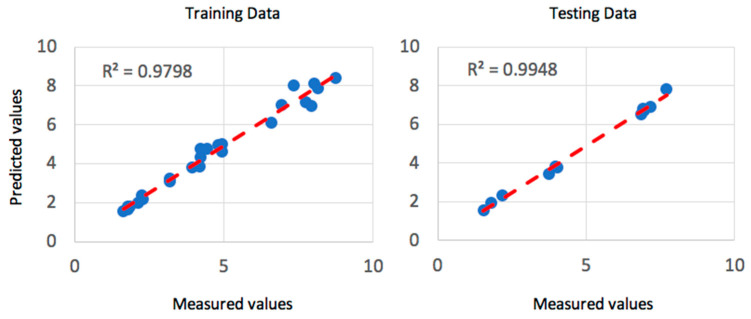
Coefficient of determination of R^2^ for the training and testing dataset of the dry turning process.

Gaussian membership functions were used with the ANFIS system for the machining parameters. Gaussian membership functions are simpler to design and faster to optimize for a small rule base [41]. The input parameters for the ANFIS system are the speed, depth of cut and the feed rate. Gaussian membership functions provide smooth output values and are illustrated in Figure 4. The speed was defined to have three levels, as in the experimental data of Table 1, of low, medium and high-speed values. Similarly, the feed rate was defined as three levels of low, medium and high feed rates. In addition, the depth of cut was assigned as three levels of shallow, medium and deep cut. The ANFIS system was simulated simultaneously with QPSO with the optimization parameters of Table 5. The training of the model was executed with 500 epochs, as shown in Figure 5 and resulted in a minimal training root mean square RMSE of 1.3 × 10^−6^ and an average testing error of 2.4% that represents a credible fit. In addition, the coefficient of determination of the training and testing datasets are presented in Figure 6 and present well fitted trend lines with high R^2^ values of 0.9798 and 0.9948 for training and testing datasets respectively.

**Figure 7:**
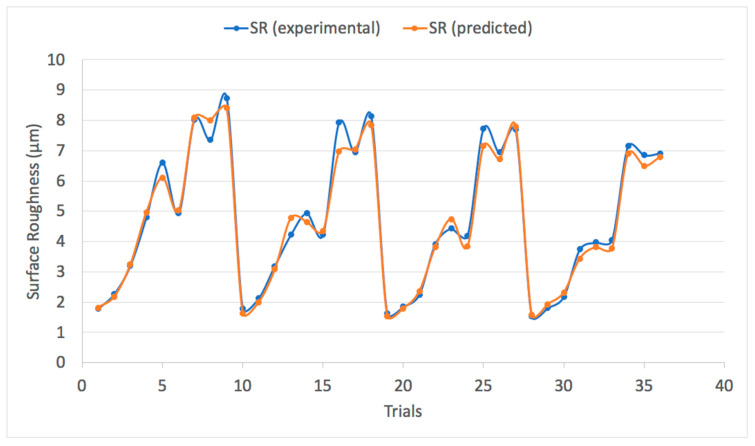
Experimental surface roughness vs. predicted surface roughness values of a dry turning process using ANFIS-QPSO.

**Figure 8:**
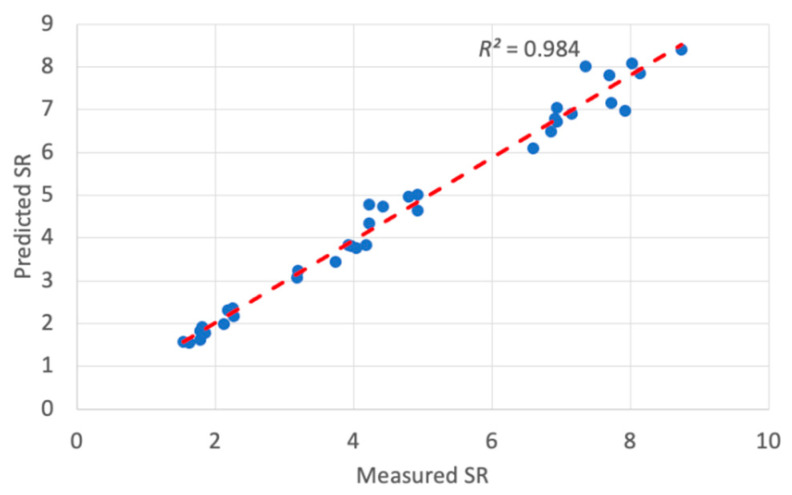
Coefficient of determination of ANFIS-QPSO prediction results for the dry turning process.

The trained ANFIS-QPSO model was then utilized to predict the optimum surface roughness of a dry turning process with the prediction results shown in Table 6. It can be noticed that the model resulted in a mean absolute percentage error (MAPE) of 4.95% that reflects the efficacy of the ANFIS-QPSO model to predict surface roughness values to a credible extent. In addition, the RMSE value was 4.86% and the coefficient of determination *R*^2^ was 0.984, which represents a good fit of the predicted values against the measured surface roughness values. Figure 7 illustrates a comparison plot between the experimental and predicted surface roughness values. ANFIS simulations were carried out to highlight the improvement of the proposed ANFIS-QPSO over the classical ANFIS system. Table 7 presents the performance criteria comparison between the classical ANFIS and ANFIS-QPSO for the dry turning process. Moreover, Figure 8 illustrates the coefficient of determination of the predicted results by ANFIS-QPSO against the measured surface roughness values. The presented results clearly highlight that ANFIS-QPSO improved the prediction accuracy of the surface roughness for the dry turning process.

**Figure 9:**
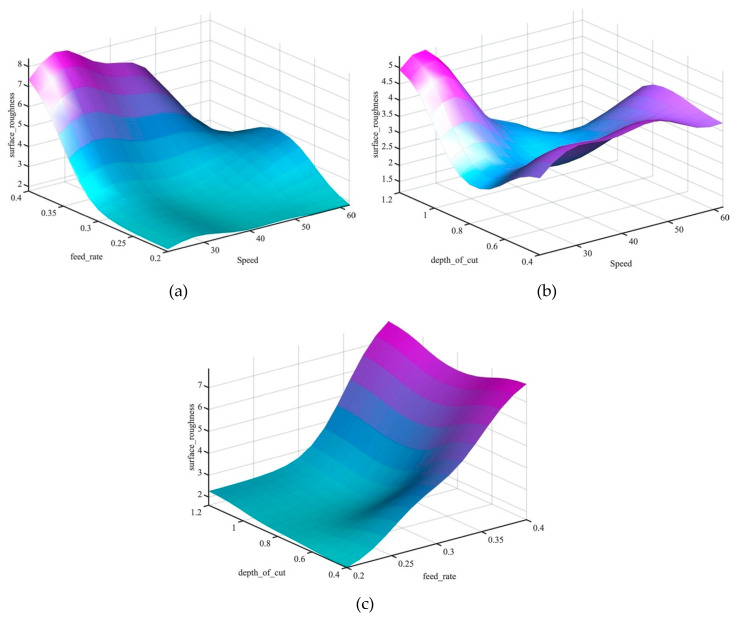
Surface plots of machining parameters vs. surface roughness of a dry turning process, (**a**) feed rate vs speed influence on surface roughness, (**b**) depth of cut vs speed influence on surface roughness, (**c**) depth of cut vs feed rate influence on surface roughness.

Figure 9 presents the 3D surface profiles of the machining parameters and their influence on the surface roughness value. The surface profile shows the interaction between the input parameters and their effect on the surface roughness value. In addition, the surface profile plot allows process operators to estimate the surface roughness value that corresponds to a given set of inputs of the feed rate, cutting speed and the depth of cut. Moreover, the surface profile plots provide the user with an estimation of optimum parameters’ values and constraints. It can be clearly observed in Figure 9a,c that the feed rate is the most prominent factor that affects the increase and decrease of the surface roughness.

In the dry turning process, increasing the feed rate increased the surface roughness due to vibrations and friction. Additionally, it can be noted that with the increase of speed the surface roughness decreased due to the decrease in built-up edge of the stainless steel [42]. Figure 9b illustrates the effectiveness of the depth of cut and cutting speed on the value of the surface roughness and revealed that the minimal surface roughness can be obtained with depth of cuts between 0.6 and 0.8 mm and cutting speeds of 30–50 m/min in a dry turning process.

### 3.2. ANFIS-QPSO for Predicting the Surface Roughness of a Cryogenic Turning Process

In a similar approach of the previous section, the ANFIS-QPSO simulation was carried out to predict the surface roughness of a cryogenic turning process. Experimental data of Table 1 were used as a training, testing and validation set for the simulation with the simulation parameters presented in Table 8.

**Figure 10:**
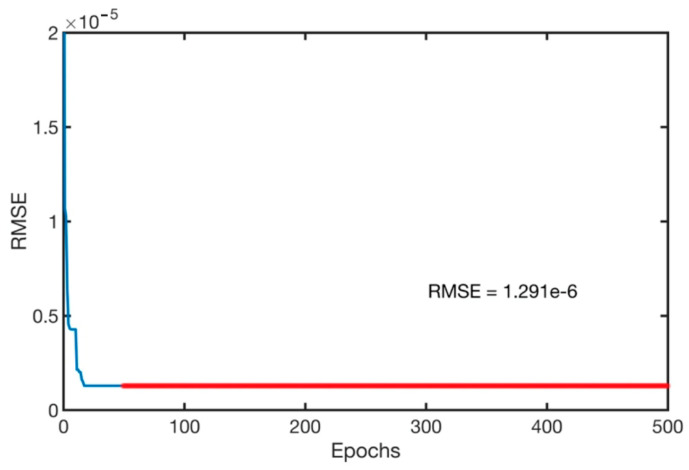
Training epochs vs. RMSE for the cryogenic turning process.

**Figure 11:**
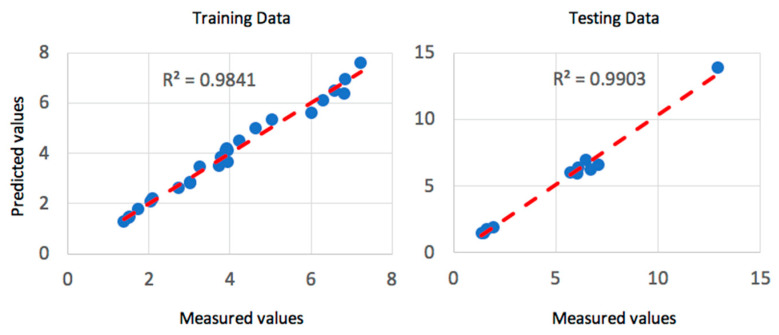
Coefficient of determination R^2^ for the training and testing dataset of the cryogenic turning process.

Training of the model was set to 500 epochs, illustrated in Figure 10, and resulted in a minimal training root mean square (RMSE) of 1.291 × 10^−3^ and an average testing error of 2.2%. Gaussian membership functions were used with the ANFIS-QPSO model as in Figure 4, and the QPSO parameters were kept constant as in the dry turning process simulation of Table 5. Figure 11 shows the coefficient of determination of the training and testing datasets in the cryogenic turning process with R^2^ values of 0.9841 and 0.9903 that correspond to a well-fitted trend line between the experimental and predicted surface roughness values.

**Figure 12:**
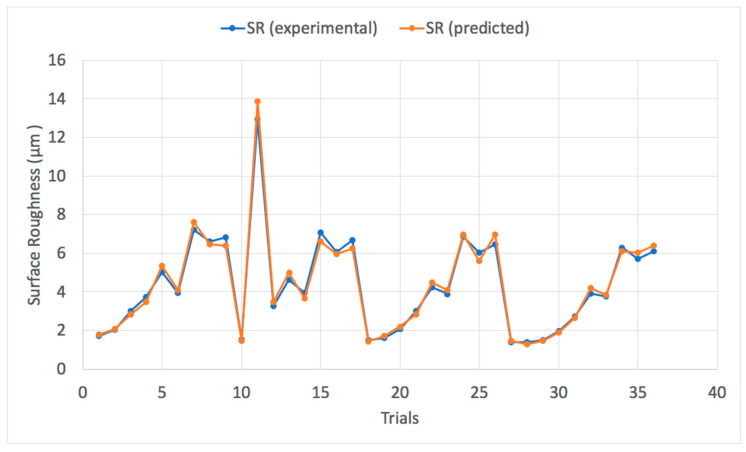
Experimental surface roughness vs. predicted surface roughness values of a cryogenic turning process using ANFIS-QPSO.

**Figure 13:**
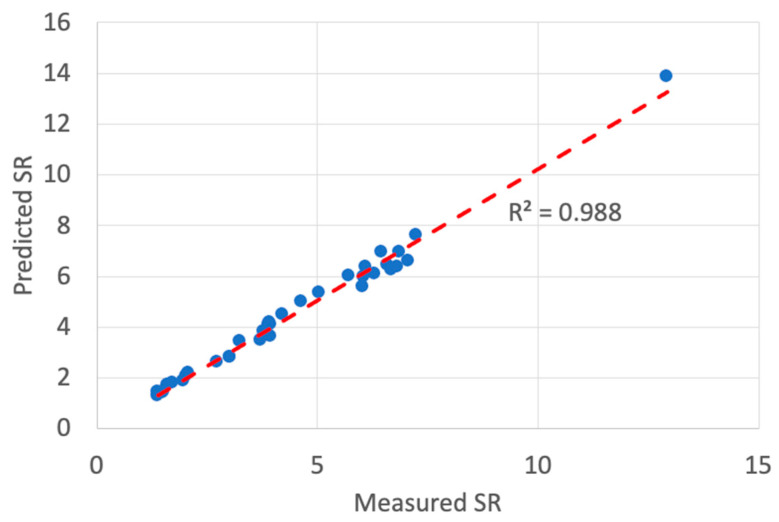
Coefficient of determination of ANFIS-QPSO prediction results for the cryogenic turning process.

The predicted values of the surface roughness for the cryogenic turning process are presented in Table 9. Figure 12 presents a comparison plot of the experimental and predicted surface roughness values of the 36 trials. The prediction MAPE was 5.15% and the RMSE was 5.08% that reflected the efficacy of the model to predict the values to a credible extent when compared with classical ANFIS as presented in Table 10. In addition, Figure 13 illustrates the coefficient of determination *R*^2^ with a value of 0.988 that presents a well fitted trend line between the predicted and the measured surface roughness values of the cryogenic turning process.

**Figure 14:**
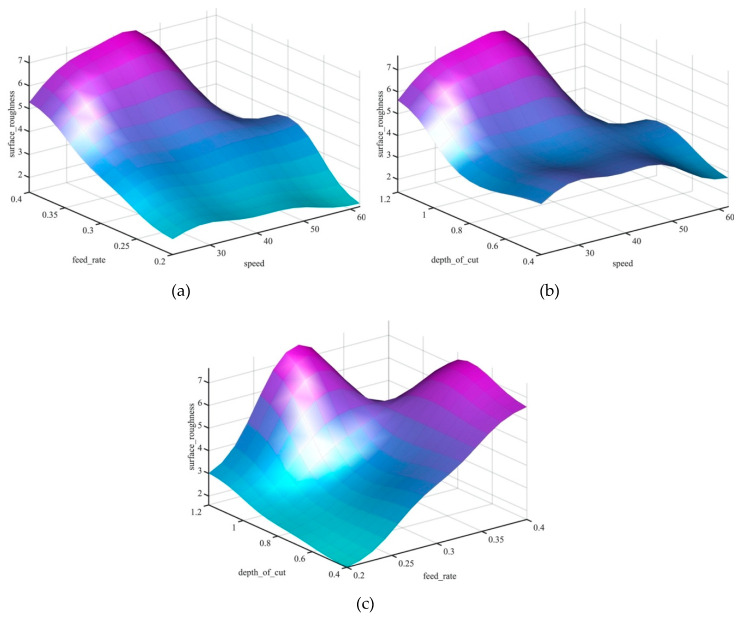
Surface plots of machining parameters vs. surface roughness of a cryogenic turning process, (**a**) feed rate vs speed influence on surface roughness, (**b**) depth of cut vs speed influence on surface roughness, (**c**) depth of cut vs feed rate influence on surface roughness.

Figure 14 shows the effectiveness of the parameters of machinery on the value of surface roughness in the cryogenic turning process. The 3D surface plot illustrates the interaction of input parameters and their effect on the surface roughness value. In the cryogenic turning process, feed rate has the most substantial effect on the surface roughness. The increase of the feed rate value increases the surface roughness. Comparing the surface roughness values of the dry and cryogenic turning process, an improvement of surface roughness values in the cryogenic process could be observed. The improvement was due to the use of fluids in the cryogenic process that lowered the temperature and improved the surface finish. Moreover, it can be noted that the lowest surface roughness value can be obtained with a feed rate of 0.2 mm and a cutting speed of 62 m/min.

## 4. Performance Comparison of ANFIS-QPSO versus Relevant Machine Learning Algorithms

In this section, the predictive accuracy of the proposed ANFIS-QPSO was compared with state of art evolutionary-optimized ANFIS algorithms that are widely used in literature such as in [43,44]. The ANFIS-QPSO performance was assessed against ANFIS-GA and ANFIS-PSO algorithms. The assessment was carried out based on the RMSE, MAPE and R^2^ values in dry and cryogenic turning processes presented in this work. The integration of ANFIS with GA and PSO falls beyond the scope of this study.

**Figure 15:**
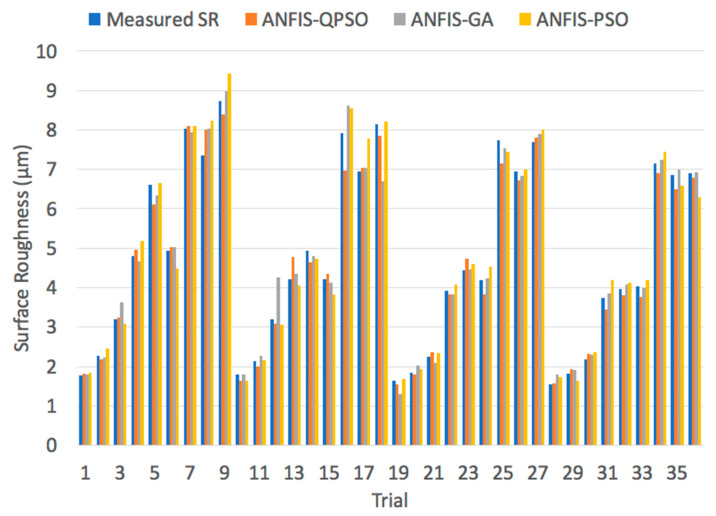
Measured surface roughness vs. predicted surface roughness values of a dry turning process using ANFIS-QPSO, ANFIS-GA and ANFIS-PSO algorithms.

Figure 15 presents a comparison between the measured and predicted surface roughness values by ANFIS-QPSO, ANFIS-GA and ANFIS-PSO algorithms for the dry turning process with the associated performance indicators presented in Table 11. All of the algorithms performed well, with slight performance measures, in predicting the surface roughness values of the dry turning process. However, it can be noted that ANFIS-QPSO had a considerably better predictive performance that outperformed ANFIS-GA and ANFIS-PSO in terms of the three performance indicators.

**Figure 16:**
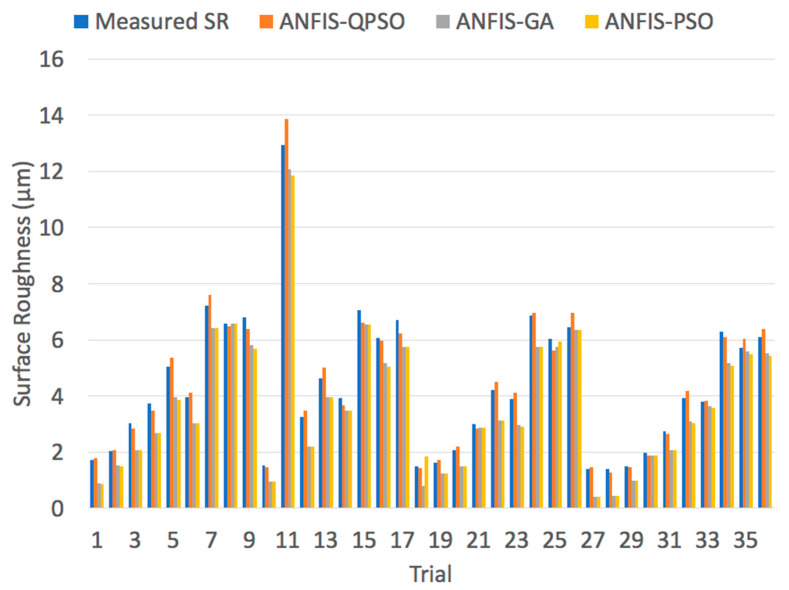
Measured surface roughness vs. predicted surface roughness values of a cryogenic turning process using ANFIS-QPSO, ANFIS-GA and ANFIS-PSO algorithms.

Similarly, for the cryogenic turning process, simulations were carried out to compare the performances of ANFIS-QPSO, ANFIS-GA and ANFIS-PSO to predict the surface roughness values. Figure 16 illustrates a comparison between the measured and predicted surface roughness values by the three algorithms. In addition, the performance indicators of the ANFIS-QPSO, ANFIS-GA and ANFIS-PSO are presented in Table 12. In contrast, ANFIS-GA had the least RMSE value in comparison with ANFIS-QPSO and ANFIS-PSO. ANFIS-QPSO outperformed ANFIS-GA and ANFIS-PSO in terms of the MAPE and R^2^ values, which was capable of reaching 5.15% and 0.988 respectively. Therefore, ANFIS-QPSO exhibited the highest prediction performance.

The superior performance of ANFIS-QPSO can be interpreted by its capability in converging to global optima solutions and avoiding local optima. The comparison results suggest that ANFIS-QPSO has a superior predictability performance in dry and cryogenic turning processes when compared with ANFIS-GA and ANFIS-PSO.

## 5. Conclusions

An ANFIS-QPSO machine learning approach was utilized in this study to predict the surface roughness of the dry and cryogenic turning process of AISI 304 stainless steel. The experimental dataset consisting of the cutting speed, feed rate and depth of cut was used to train the ANFIS-QPSO system. ANFIS-QPSO combines the model-free characteristics of fuzzy systems with the strength of quantum-inspired optimization in terms of faster convergence and robustness. These characteristics are important to estimate the surface roughness with a given set of machine parameters and thus saving the cost and time of experimental trials. The predicted surface roughness values were matched with the measured values in order to demonstrate the efficacy of the ANFIS-QPSO. The predicted outcomes were found to be in close agreement to the experimental values. In the dry turning process, the MAPE between experimental and predicted surface roughness values was 4.95%. While for the cryogenic turning process the MAPE was 5.15%. A comparison of prediction accuracy between ANFIS, ANFIS-GA, ANFIS-PSO and the proposed ANFIS-QPSO was carried out and shows that the ANFIS-QPSO resulted in greater accuracy in terms of the MAPE, RMSE and R^2^ values for both dry and cryogenic turning processes.

## Figures and Tables

**Table 1:** Nomenclature.

$R_{a}$	Arithmetic Surface Roughness (µm)
$R_{t}$	Maximum peak to valley height (µm)
V	Cutting Speed (m/min)
f	Feed Rate (mm/rev)
d	Depth of Cut (mm)
$r_{2}$	Tool Nose Radius
α	major cutting edge angles
β	end cutting edge angle

**Table 2:** Cryogenic turning process experimental data [40].

Trial	Speed (m/min)	Feed Rate (mm/rev)	Depth of Cut (mm)	SR (Experimental)
1	22	0.2	0.4	1.72
2	22	0.2	0.8	2.03
3	22	0.2	1.2	3.02
4	22	0.3	0.4	3.72
5	22	0.3	0.8	5.04
6	22	0.3	1.2	3.95
7	22	0.4	0.4	7.23
8	22	0.4	0.8	6.59
9	22	0.4	1.2	6.81
10	22	0.2	0.4	1.52
11	31	0.2	0.8	12.93
12	31	0.2	1.2	3.25
13	31	0.3	0.4	4.64
14	31	0.3	0.8	3.93
15	31	0.3	1.2	7.07
16	31	0.4	0.4	6.06
17	31	0.4	0.8	6.69
18	31	0.4	1.2	1.49
19	44	0.2	0.4	1.61
20	44	0.2	0.8	2.08
21	44	0.2	1.2	3.01
22	44	0.3	0.4	4.22
23	44	0.3	0.8	3.89
24	44	0.3	1.2	6.85
25	44	0.4	0.4	6.02
26	44	0.4	0.8	6.46
27	44	0.4	1.2	1.38
28	62	0.2	0.4	1.38
29	62	0.2	0.8	1.5
30	62	0.2	1.2	1.97
31	62	0.3	0.4	2.73
32	62	0.3	0.8	3.92
33	62	0.3	1.2	3.78
34	62	0.4	0.4	6.3
35	62	0.4	0.8	5.72
36	62	0.4	1.2	6.1

**Table 3:** Dry turning process experimental data [40].

Trial	Speed (m/min)	Feed Rate (mm/rev)	Depth of Cut (mm)	SR (Experimental)
1	22	0.2	0.4	1.78
2	22	0.2	0.8	2.27
3	22	0.2	1.2	3.2
4	22	0.3	0.4	4.8
5	22	0.3	0.8	6.6
6	22	0.3	1.2	4.93
7	22	0.4	0.4	8.03
8	22	0.4	0.8	7.35
9	22	0.4	1.2	8.74
10	22	0.2	0.4	1.79
11	31	0.2	0.8	2.13
12	31	0.2	1.2	3.19
13	31	0.3	0.4	4.22
14	31	0.3	0.8	4.93
15	31	0.3	1.2	4.22
16	31	0.4	0.4	7.92
17	31	0.4	0.8	6.94
18	31	0.4	1.2	8.14
19	44	0.2	0.4	1.63
20	44	0.2	0.8	1.85
21	44	0.2	1.2	2.25
22	44	0.3	0.4	3.92
23	44	0.3	0.8	4.43
24	44	0.3	1.2	4.19
25	44	0.4	0.4	7.73
26	44	0.4	0.8	6.94
27	44	0.4	1.2	7.7
28	62	0.2	0.4	1.54
29	62	0.2	0.8	1.81
30	62	0.2	1.2	2.18
31	62	0.3	0.4	3.74
32	62	0.3	0.8	3.97
33	62	0.3	1.2	4.04
34	62	0.4	0.4	7.15
35	62	0.4	0.8	6.85
36	62	0.4	1.2	6.91

**Table 4:** ANFIS simulation parameters—dry turning process.

ANFIS Simulation Parameters—Dry Turning Process
Fuzzy system: Takagi-Sugeno fuzzy system
Training set = 70% of the data
Testing set = 15% of the data
Validation set = 15% of the data
Training epochs = 500
Fuzzy Membership functions type: Gaussian Membership functions
Number of nonlinear parameters: 18
Number of linear parameters: 108
Number of nodes: 78
Number of fuzzy rules: 27
Number of checking data pairs: 0
Number of training data pairs: 16
Total number of parameters: 126

**Table 5:** QPSO simulation parameters with the ANFIS system for the dry turning process.

Parameter	Value
Number of iterations	800
Particle population	100
Cognitive acceleration: *c_1_*	2
Social coefficient: *c_2_*	2
Contraction-expansion factor: β	0.85

**Table 6:** ANFIS-QPSO prediction results of the surface roughness values for the dry turning process.

Trial	Speed (m/min)	Feed Rate (mm/rev)	Depth of Cut (mm)	SR (Experimental)	SR (Predicted)	Error %
1	22	0.2	0.4	1.78	1.82	2.25%
2	22	0.2	0.8	2.27	2.18	3.96%
3	22	0.2	1.2	3.2	3.24	1.25%
4	22	0.3	0.4	4.8	4.97	3.54%
5	22	0.3	0.8	6.6	6.10	7.58%
6	22	0.3	1.2	4.93	5.02	1.83%
7	22	0.4	0.4	8.03	8.09	0.75%
8	22	0.4	0.8	7.35	8.00	8.84%
9	22	0.4	1.2	8.74	8.40	3.89%
10	22	0.2	0.4	1.79	1.63	8.94%
11	31	0.2	0.8	2.13	2.00	6.10%
12	31	0.2	1.2	3.19	3.08	3.30%
13	31	0.3	0.4	4.22	4.77	13.00%
14	31	0.3	0.8	4.93	4.64	5.93%
15	31	0.3	1.2	4.22	4.35	3.03%
16	31	0.4	0.4	7.92	6.97	12.00%
17	31	0.4	0.8	6.94	7.03	1.35%
18	31	0.4	1.2	8.14	7.85	3.59%
19	44	0.2	0.4	1.63	1.54	5.50%
20	44	0.2	0.8	1.85	1.79	3.13%
21	44	0.2	1.2	2.25	2.36	4.98%
22	44	0.3	0.4	3.92	3.83	2.35%
23	44	0.3	0.8	4.43	4.74	6.93%
24	44	0.3	1.2	4.19	3.84	8.34%
25	44	0.4	0.4	7.73	7.16	7.34%
26	44	0.4	0.8	6.94	6.72	3.10%
27	44	0.4	1.2	7.7	7.80	1.34%
28	62	0.2	0.4	1.54	1.57	1.69%
29	62	0.2	0.8	1.81	1.93	6.48%
30	62	0.2	1.2	2.18	2.32	6.63%
31	62	0.3	0.4	3.74	3.44	8.00%
32	62	0.3	0.8	3.97	3.81	4.10%
33	62	0.3	1.2	4.04	3.77	6.67%
34	62	0.4	0.4	7.15	6.90	3.53%
35	62	0.4	0.8	6.85	6.49	5.30%
36	62	0.4	1.2	6.91	6.79	1.80%

**Table 7:** ANFIS vs. ANFIS-QPSO performance comparison for the dry turning process.

Criteria	ANFIS	ANFIS-QPSO
*RMSE*	5.3%	4.86%
*MAPE*	5.47%	4.95%
*R* ^2^	0.972	0.984

**Table 8:** ANFIS simulation parameters—cryogenic turning process.

ANFIS Simulation Parameters—Cryogenic Turning Process
Fuzzy system: Takagi-Sugeno fuzzy system
Training set = 70% of the data
Testing set = 15% of the data
Validation set = 15% of the data
Training epochs = 500
Fuzzy Membership functions type: Gaussian Membership functions
Number of nodes: 58
Number of linear parameters: 72
Number of nonlinear parameters: 24
Total number of parameters: 96
Number of training data pairs: 30
Number of checking data pairs: 0
Number of fuzzy rules: 18

**Table 9:** ANFIS-QPSO prediction results of the surface roughness values for a cryogenic turning process.

Trial	Speed (m/min)	Feed Rate (mm/rev)	Depth of Cut (mm)	SR (Experimental)	SR (Predicted)	Error %
1	22	0.2	0.4	1.72	1.79	4.12%
2	22	0.2	0.8	2.03	2.07	2.15%
3	22	0.2	1.2	3.02	2.84	5.93%
4	22	0.3	0.4	3.72	3.49	6.28%
5	22	0.3	0.8	5.04	5.36	6.44%
6	22	0.3	1.2	3.95	4.10	3.75%
7	22	0.4	0.4	7.23	7.61	5.22%
8	22	0.4	0.8	6.59	6.47	1.80%
9	22	0.4	1.2	6.81	6.39	6.19%
10	22	0.2	0.4	1.52	1.47	3.17%
11	31	0.2	0.8	12.93	13.88	7.38%
12	31	0.2	1.2	3.25	3.46	6.32%
13	31	0.3	0.4	4.64	5.00	7.74%
14	31	0.3	0.8	3.93	3.66	6.91%
15	31	0.3	1.2	7.07	6.62	6.42%
16	31	0.4	0.4	6.06	5.97	1.52%
17	31	0.4	0.8	6.69	6.24	6.74%
18	31	0.4	1.2	1.49	1.43	4.07%
19	44	0.2	0.4	1.61	1.73	7.62%
20	44	0.2	0.8	2.08	2.20	5.66%
21	44	0.2	1.2	3.01	2.82	6.17%
22	44	0.3	0.4	4.22	4.50	6.70%
23	44	0.3	0.8	3.89	4.10	5.35%
24	44	0.3	1.2	6.85	6.96	1.56%
25	44	0.4	0.4	6.02	5.61	6.84%
26	44	0.4	0.8	6.46	6.96	7.81%
27	44	0.4	1.2	1.38	1.46	5.90%
28	62	0.2	0.4	1.38	1.28	7.42%
29	62	0.2	0.8	1.5	1.45	3.30%
30	62	0.2	1.2	1.97	1.89	3.88%
31	62	0.3	0.4	2.73	2.64	3.13%
32	62	0.3	0.8	3.92	4.19	6.97%
33	62	0.3	1.2	3.78	3.84	1.68%
34	62	0.4	0.4	6.3	6.10	3.16%
35	62	0.4	0.8	5.72	6.03	5.38%
36	62	0.4	1.2	6.1	6.38	4.64%

**Table 10:** ANFIS vs. ANFIS-QPSO performance comparison for the cryogenic turning process.

Criteria	ANFIS	ANFIS-QPSO
*RMSE*	5.51%	5.08%
*MAPE*	5.63%	5.15%
*R* ^2^	0.981	0.988

**Table 11:** Performance comparison of ANFIS-QPSO, ANFIS-GA and ANFIS-PSO for the dry turning process.

Criteria.	ANFIS-QPSO	ANFIS-GA	ANFIS-PSO
*RMSE*	**4.86%**	5.06%	5.23%
*MAPE*	**4.95%**	5.13%	5.29%
*R* ^2^	**0.984**	0.9819	0.9822

**Table 12:** Performance comparison of ANFIS-QPSO, ANFIS-GA and ANFIS-PSO for the cr+yogenic turning process.

Criteria.	ANFIS-QPSO	ANFIS-GA	ANFIS-PSO
*RMSE*	5.08%	**5.02%**	5.46%
*MAPE*	**5.15%**	5.34%	5.53%
*R* ^2^	**0.988**	0.9807	0.9742
